# Molecular docking of polyphenol compounds and exploring the anticoagulant activity of *Costus speciosus* extracts *in vitro* and *in vivo*

**DOI:** 10.1016/j.toxrep.2025.101961

**Published:** 2025-02-19

**Authors:** Sara Gheraibia, Noureddine Belattar, Marwa E. Hassan, Aziza A. El-Nekeety, Eslam R. El-Sawy, Mosaad A. Abdel-Wahhab

**Affiliations:** a Laboratory of Applied Biochemistry, Faculty of Sciences of Nature and Life, Ferhat Abbes University, Setif 1, Algeria; b Toxicology Department, Research Institute of Medical Entomology, Giza, Egypt; c Food Toxicology & Contaminants Department, National Research Centre, Dokki, Cairo, Egypt; d Chemistry of Natural Products Department, National Research Centre, Dokki, Cairo, Egypt

**Keywords:** Anticoagulant activity, *Costus speciosus*, *Costaceae*, Phenolic compounds, Molecular docking, Thrombin inhibitor complex (PDB: 1KTS)

## Abstract

Anticoagulants have an important role in the prevention of cardiovascular disorders. *Costus speciosus* (*Costaceae*) is a medicinal herb used to treat COVID-19-induced thrombosis. The purpose of this study was to assess the anticoagulant activity of various *C. speciosus* aqueous (CSAE), ethanol (SCEE), and methanol (CSME) extracts in *vivo* and in *vitro* utilizing thrombin time (TT), activated partial thromboplastin time (aPTT), and prothrombin time (PT). Different concentrations of the three extracts were used to evaluate the anticoagulation effects *in vitro*. In the *in vivo* assay, male Sprague Dawley rats were used to test the CSME as *in vivo* anticoagulants. Three groups of rats included the control group and the groups that received CSME daily at a low (200 mg/kg) or high dose (400 mg/kg b.w) for 2 weeks. The molecular docking of the major bioactive constituents of the methanolic extract against the binding site of the thrombin inhibitor complex was evaluated. The HPLC detected 13, 10 and 11 polyphenols in the methanolic, ethanolic and aqueous extracts, respectively. The *in vitro* results showed that all the studied extracts had anticoagulant activity and increased aPTT, TT, and PT time. The *in vivo* experiment supported the *in vitro* results and demonstrated that CSME greatly prolonged the anticoagulant characteristics when compared to the negative control. Both findings suggested that these extracts have significant anticoagulant activity, with CSME being more effective and potentially useful in pharmaceutical applications as a natural anticoagulant medication.

## Highlights

• *Costus speciosus* is rich in polyphenols with different pharmacological activities.
• Different extracts of this plant showed potent anticoagulant activity *in vitro.*
• The extracts prolonged the anticoagulant indices (PT, TT, and aPTT) *in vitro.*
• The anticoagulant activity of the methanol extract was further tested *in vivo.*
• The methanol extract may be promising for the development of natural anticoagulants.

## Graphical Abstract

**Figure:**
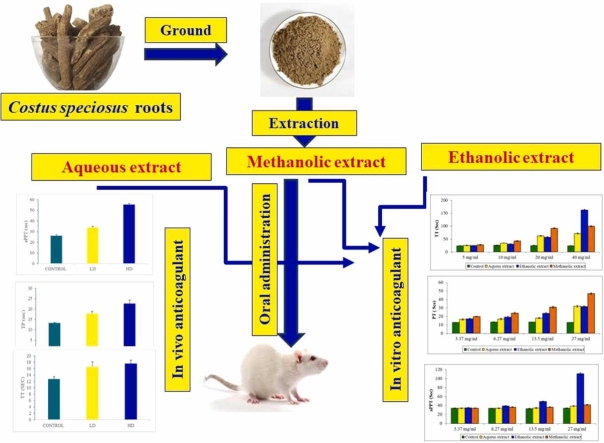

## 1 Introduction

The Coronavirus Disease 2019 (COVID-19) pandemic was linked to acute inflammation and organ damage [1] and produces considerable coagulation activity, leading to thrombosis and extensive microangiopathy [2], [3], [4]. According to various publications, coagulation disorders have been linked to a higher mortality risk [5], [6], [7]. Thromboembolism is a serious vascular illness worldwide and is the 3rd greatest cause of vascular disease, with an incidence of 1–2 cases per 1000 individuals globally each year [8]. The coagulation process is the consequence of consecutive processes in which thrombin, as a final enzyme, transform soluble fibrinogen into insoluble fibrin [9]. Thrombosis happens when the blood components create an unnatural mass called a thrombus, which inhibits a living animal's circulatory system [10].

Anti-coagulant chemicals used to minimize or prevent the development of blood clots *in vitro* include heparin, EDTA, sodium citrate (3.8 %), and a combination of potassium and ammonium oxalate [11]. These anti-coagulants are generated synthetically; the disadvantages of this with existing thrombolytic therapy include slow and incomplete thrombolysis and frequent bleeding problems [9]. For example, long-term usage of heparin causes bleeding of blood arteries in the brain as well as thrombocytopenia [12]. Furthermore, the cost of these compounds is quite high, so there is an urgent need to produce natural anticoagulants from plants to lessen side effects at accessible prices [13].

*Costus speciosus* (family *Costaceae*) is a well-known herbaceous plant that grows widely in Asian countries, including India, Sri Lanka, Indonesia, and Malaysia [14]. The extracts from different parts of this plant have shown several pharmacological activities. Several solvent extracts of this plant's root have demonstrated strong anticancer activity against several cancer types in a dose-dependent manner [15], [16]. In addition to many pharmacological activities such as antidiabetic [17], antioxidant [18], anti-arthritic [19], anti-inflammatory [20], antipyretic [21], and hepatoprotective [22]. Previous research has revealed that the concentration of plant polyphenols varies with extraction solvents and extraction conditions, affecting the biological activity of the extract [23]. Molecule docking has become an important step in drug development since it simulates the bonding forms between compounds and target enzymes [24], [25]. The purpose of this study was to determine the anticoagulant properties of aqueous, ethanolic, and methanolic extracts of *Costus speciosus* (*C. speciosus*) *in vitro* and *in vivo*.

## 2 Material and methods

### 2.1 Plant material

The roots of *C. speciosus* were obtained from local herbal stores in Setif, Algeria. A plant taxonomist from the Botany Department, Faculty of Natural and Life Sciences, University of Ferhat Abbas Setif 1, Algeria, has certified the plant and a voucher specimens voucher specimen was deposited under the number: AM/SC0081/22 at the Herbarium of University of Ferhat Abbas Setif 1, Algeria

### 2.2 Plant extraction and HPLC analysis

The roots of the plant were washed and dried in the oven at 40 °C for one week until their weight was constant. The dried roots were crushed to a particle size of 200–500 μm and extracted with water, 70 % ethanol, or 70 % methanol at room temperature under agitation for 36 h. The extracts were evaporated under reduced pressure and the final residue was kept as a powder at 4 °C until employed in subsequent experiments. The total phenolic contents were determined by an Agilent 1260 series HPLC as detailed in our previous work [16].

### 2.3 In vitro anticoagulant evaluation

The effect of different concentrations of *C. speciosus* aqueous (CSAE), ethanolic (CSEE), and methanolic (CSME) extracts on the coagulation activity was examined using prothrombin time (PT), thrombin time (TT), and activated partial thromboplastin time (aPTT) utilizing a hemostasis analyzer (Stago-STA Compact Max®). A sample of each extract was dissolved in a physiological serum and all analyses were performed three times. A venous puncture was used to collect approximately 10 ml of blood from healthy volunteers of both sexes (ages 18–35 years old) at the University Hospital Center, Saadna Abedenour, Steif, Algeria, who had not taken any medications for at least one week prior to the blood collection. The donors provided informed consent, and the protocol was permitted and accepted by the ethics committee of Ferhat Abbes University, Steif 1, Algeria (ethical approval # D01N01UN190120120005). To prevent natural coagulation, 9 μl of blood was mixed with 1 μl of 3.8 % trisodium citrate solution. The blood samples were immediately centrifuged for 15 min at 3000 rpm in a cooled centrifuge, and platelet-poor plasma (PPP) was separated and stored at −4 °C until needed.

#### 2.3.1 Prothrombin time (PT) assay

The PT test revealed the action in the extrinsic route, and the test was carried out using commercial reagent kits (Biolabo S-F2160 France). A volume of plasma (90 μl) was mixed with 10 μl of the extract and incubated for 5 min at 37 °C. The clotting time was measured using a digital coagulometer after adding 200 μl of pre-warmed PT assay reagent (calcium chloride and rabbit brain extract) that had been pre-warmed at 37 °C for 10 min. Plasma alone (with vehicle only) served as a control (no anticoagulant activity).

#### 2.3.2 Activated partial thromboplastin time (aPPT) assay

The aPTT is often referred to as KCCT (Kaolin Cephalin Clotting Time) or PTK, and it was used to evaluate the action of both common and intrinsic routes. The test was performed with commercial reagent kits (Biolabo SA FO2160, France). Plasma (90 μl) was mixed with 10 μl of the extracts and incubated at 37 °C for 5 min, before adding prewarmed aPTT reagent. After 2 min of incubation, 25 mM pre-warmed (37 °C) calcium chloride was added. A digital coagulometer was used to measure clotting time, and plasma (only with a vehicle) served as a control (no anticoagulant activity).

#### 2.3.3 Thrombin time (TT)

This assay evaluates the duration of thrombin production. A 130 μl of human plasma (pre-incubated at 37 °C for 5 min before usage) was incubated with different doses of each extract for 5 min at 37 °C. Normal plasma was used as the control. To initiate the reaction, each sample received a predetermined concentration (150 μl) of bovine thrombin (2.5 U/ml, Sigma), and the time for colt formation was recorded. The results were expressed in seconds as a prolongation time relative to the controls.

### 2.4 *In vivo* estimation of the CSME

#### 2.4.1 Animals

The Animal House Colony, National Research Centre (NRC), Dokki, Cairo, Egypt, donated thirty male Sprague Dawley rats weighing between 150 and 160 g. The animals were fed a conventional lab diet (metabolizable energy: 12.08 MJ; fat: 36.3; protein: 160.4; fiber: 41 g/kg provided by Meladco Feed Co., Aubor City, Cairo, Egypt). At the Animal House Lab., NRC, animals were housed in polycarbonate cages in a temperature-controlled (25 ± 1 °C), humidity-controlled (50 ± 5 %), 12-h dark/light cycle, and an environment free of chemical contamination. All animal experiments followed the ARRIVE guidelines and were carried out in accordance with the National Institutes of Health guide for the care and use of laboratory animals (NIH Publications No. 8023, revised 1978), and the protocol was permitted and approved by the NRC's Animal Care and Use Committee (ethical approval number 13050302/2022).

#### 2.4.2 Experimental design

The animals were randomly assigned to three groups of ten animals each and treated orally for two weeks, including an untreated control group (rats were orally administered a dose of physiological saline and served as negative controls), and the groups received either low (200 mg/kg b.w) or high (400 mg/kg b.w) doses of the CSME. At the conclusion of the trial, blood samples were drawn from each rat under isoflurane anesthesia via the retro-orbital venous plexus. The blood samples were immediately combined with 3.2 % trisodium citrate in a 9:1 vol ratio, and the combination was centrifuged for 20 min at 4000 rpm to obtain citrated platelet-poor plasma (PPP). Each citrated PPP was immediately transferred to a plastic tube, and the clotting time was measured using the same protocols as the *in vitro* assay.

#### 2.4.3 Molecular docking

The CSME components were docked against the thrombin inhibitor complex (PDB: 1KTS) using PyRx tools Autodock Vina (version 1.1.2), as described by Stanzione et al. [24]. The target thrombin protein structure (PDB code: 1KTS) was retrieved from the PDB database (accessed on November 5, 2024). The initial ligand and water molecules were removed from the protein using the VEGA ZZ 2.3.2 tool, followed by the addition of polar hydrogen and Kollman charges, and finally converted to PDBQT format using Autodock Vina tools [24]. The original ligand (dabigatran, C24) was re-docked to ensure docking accuracy. The original ligand fundamental pose was used as a reference to validate the simulation procedure and determine grid box dimensions and positioning. Furthermore, all molecules (CSME components) were created with ChemDraw Ultra 10.0 and saved as mol files, which were then protonated, minimized, and converted to pdb files using Open Babel software. The resulting pdb file was submitted to Autodock Vina tools for torsion number setting and pdbqt file creation. The grid map was created using Auto-Grid using a grid box, and the values recorded were X = 16.85, Y= -14.27, and X = 19.63. The number of docked poses created for each chemical at the active pocket was ten, and they were ranked according to the binding energy. The pose with the lowest binding energy and 0 Å root-mean-square deviation (RMSD) was considered to be the fitted and complexed with receptor for analysis. The molecular interactions and binding models of the top poses were visually examined with BIOVIA Discovery Studio 2021.

### 2.5 Statistical analysis

The results are shown as the mean ± SD of three replicates for each sample. The data were statistically evaluated using analysis of variance (ANOVA) with Graph Pad Prism's general linear model technique. Duncan's multiple range tests were performed to determine the significance of individual groups at p ≤ 0.05.

## 3 Results

### 3.1 HPLC analysis

In a previous work, we reported that the HPLC analysis revealed the presence of 13 chemicals in the methanolic extract, only 10 in the ethanolic extract, and 11 in the aqueous extract (Table 1S Supplementary materials). The methanolic extract included the following polyphenols: chlorogenic acid (1175.07 µg/g), naringenin (1069.65 µg/g), gallic acid (689.73 µg/g), pyrocatechol (371.77 µg/g), ellagic acid (365.48 µg/g), and syringic acid (136.22 µg/g). Quercetin, coumaric acid, 4′.7-dihydroxyiso flavone, pyronyl gallate, caffeic acid, cinnamic acid, and vanillin were found in lower amounts (127.65, 125.92, 100.54, 93.79, 92.86, 49.51, and 40.29 µg/g, respectively). The ethanolic extract lacked quercetin, vanillin, and cinnamic acid, but was high in chlorogenic acid (1206.48 µg/g), naringenin (1025.17 µg/g), gallic acid (747.58 µg/g), and pyrocatechol (300.34 µg/g). The aqueous extract lacked coumaric acid and vanillin but had high levels of gallic acid (2470.21 µg/g), naringenin (873.80 µg/g), chlorogenic acid (590.55 µg/g), and pyro catechol (306.19 µg/g). Quercetin, caffeic acid, and ellagic acid were found at lower amounts (172.35, 154.29, and 139.69 µg/g, respectively).

### 3.2 Anticoagulant activity

Anticoagulant activity is typically assessed using aPTT, PT, and TT activities as standard coagulation tests, which are generally associated with the intrinsic and extrinsic routes of the coagulation process. The study found significant (P < 0.05) lengthening of aPTT with the extracts at all tested doses compared to the control. The concentration of 27 mg/ml produced the longest aPTT durations when compared to the other concentrations tested (Table 1). The aPTT times for the control, SCAE, ACEE, and SCME were 34.6, 36.5, 36.5, and 111.6 s, respectively.

**Table 1:** Activated partial thromboplastin time (s) of various extracts of *C. speciosus* (mean of three replicates ± SD).

Treatments Concentration	Control	SCAE	SCEE	SCME
3.37 mg/ml	34.00 ± 0.9^a^	33.50 ± 0.8^a^	33.00 ± 0.8^a^	34.00 ± 0.8^a^
6.27 mg/ml	34.00 ± 0.8^a^	34.00 ± 0.92^a^	34.40 ± 0.93^a^	38.80 ± 0.94^b^
13.5 mg/ml	35.00 ± 0.8^a^	39.00 ± 1.00^b^	40.00 ± 1.01^c^	41.00 ± 2.31^c^
27 mg.ml	34.60 ± 0.7^a^	36.50 ± 0.87^a^	36.40 ± 0.77^a^	111.60 ± 0.88^B^

Within each row, means superscript with different letter are significantly different (P < 0.05).

The current results demonstrated significant differences (P < 0.05) in the prolonging of TP with the various extracts at all tested concentrations compared to the control (Table 2). The data showed that increasing extract concentration lengthened TP duration for all three extracts, with the exception of SCME at 6.27 and 13.5 mg/ml, which did not differ substantially. The longest TP durations were obtained at a concentration of 27.0 mg/ml for all extracts, with values of 20.0, 24.5, 31.0, and 47.0 for the control, SCAE, SCEE, and SCME. These results showed that SCME was the best extract, with the longest TP duration.

**Table 2:** Prothrombin Time (s) of various extracts of *C. speciosus* (mean of three replicates ± SD).

Treatments Concentration	Control	SCAE	SCEE	SCME
3.37 mg/ml	13.00 ± 0.10^a^	13.50 ± 0.80^a^	13.50 ± 0.40^a^	13.00 ± 0.30^a^
6.27 mg/ml	16.50 ± 0.10^a^	17.10 ± 0.90^a^	18.10 ± 0.90^b^	31.10 ± 0.84^c^
13.5 mg/ml	17.20 ± 0.02^a^	19.00 ± 0.70^a^	23.60 ± 1.02^b^	32.17 ± 0.90^c^
27 mg.ml	20.00 ± 0.06^a^	24.50 ± 1.00^b^	31.00 ± 1.01^c^	47.00 ± 0.90^d^

Within each row, means superscript with different letter are significantly different (P < 0.05).

Table (3) shows substantial differences (P < 0.05) in TT time between the three extracts at all tested doses and the control. The longest TT times were reported at 40.0 mg/ml compared to the other tested concentrations; however, the TT time for SCME at 20 mg/ml was longer than at 40 mg/ml. At 40 mg/ml, the TT time was 28.0, 43.0, 92.0, and 162.0 s for the control, SCAE, SCEE, and SCME, respectively.

**Table 3:** Thrombin time (s) of various extracts of *C. speciosus* (mean of three replicates ± SD).

Treatments Concentration	Control	SCAE	SCEE	SCME
5 mg/ml	24.00 ± 0.80^a^	26.00 ± 0.90^a^	25.00 ± 0.90^a^	24.00 ± 0.80^a^
10 mg/ml	25.15 ± 0.90^a^	34.45 ± 0.10^b^	62.15 ± 0.10^c^	71.70 ± 1.40^d^
20 mg/ml	24.50 ± 0.90^a^	30.75 ± 1.60^b^	57.45 ± 1.60^c^	100.70 ± 1.80^d^
40 mg.ml	28.00 ± 1.00^a^	43.00 ± 1.90^b^	92.00 ± 1.90^c^	162.00 ± 2.00^d^

Within each row, means superscript with different letter are significantly different (P < 0.05).

**Fig. 1:**
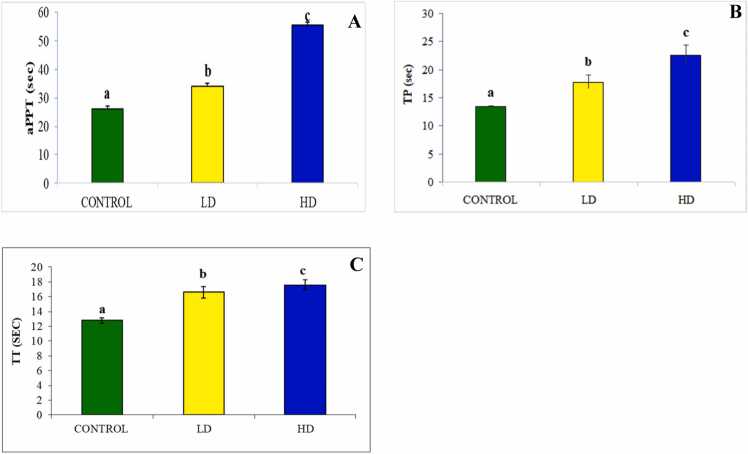
*In vivo* Anticoagulant activity of SCME evaluated by the measurement of **(A)** aPTT, **(B)** TP, and **(C)** TT. The data are expressed as means ± SD. The column superscripted with different letter (a, b and c) is significantly different (P < 0.05).

The SCME's current *in vitro* anticoagulant results were encouraging, so we decided to investigate its activity in *vivo* to determine its true effect on the organism. The results showed that the in *vivo* aPTT, TT, and PT were concentration-dependent. The observed values of aPTT were 34.0 and 55.4 s at the low dose (200 mg/kg b.w) and high dose (400 mg/kg b.w), respectively, as opposed to the control, which recorded 26.2 s (Fig. 1A). Furthermore, as the dose of the extract increased, so did the measured TP time. The recorded values for the low and high doses were 17.8 and 2.6 s, respectively, while the recorded TP time for the control was 13.4 s (Fig. 1B). The same elongation tendency was observed for TT, which was greater in the group that received the high dose (Fig. 1C), with recorded values of 16.6 and 17.6 s in the low and high-dose-treated groups, respectively. However, the TT time for the control group was 12.8 s.

### 3.3 Molecular docking

**Fig. 2:**
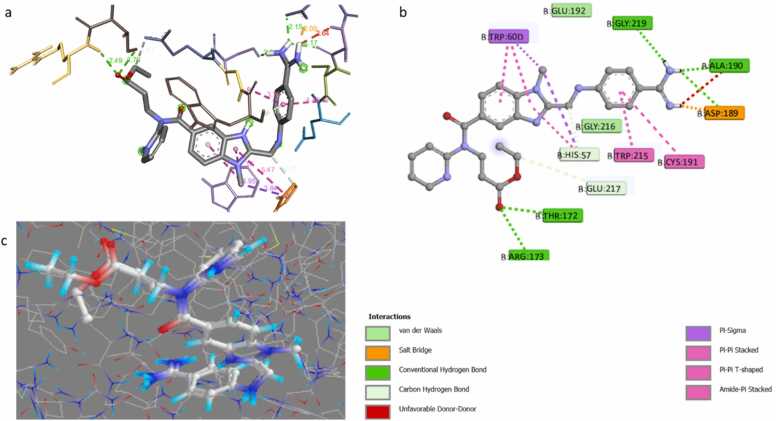
(a) the 3D binding mode of the original ligand (dabigatran) within active pocket of the thrombin inhibitor complex (PDB: 1KTS); (b) the original ligand (dabigatran) within the active pocket of the thrombin inhibitor complex (PDB: 1KTS); and (c) the original ligand and the re-docked original ligand inside the active pocket of the thrombin inhibitor complex (PDB: 1KTS).

The molecular docking study of the CSME components towards the thrombin inhibitor complex (PDB: 1KTS) observed that the original ligand (C24) of the target thrombin protein complex revealed a docking score (S) of −9.4 kcal/mol. Furthermore, the C24 exhibited hydrophilic interactions (hydrogen bonds) with the critical amino acids of the target thrombin protein cave, including ASP189, GLU192, GLY219, ALA190, GLY216, HIS57, GLU217, THR172, and ARG173, as well as Pi-Pi and Pi-alkyl interactions (Fig. 2).

**Fig. 3:**
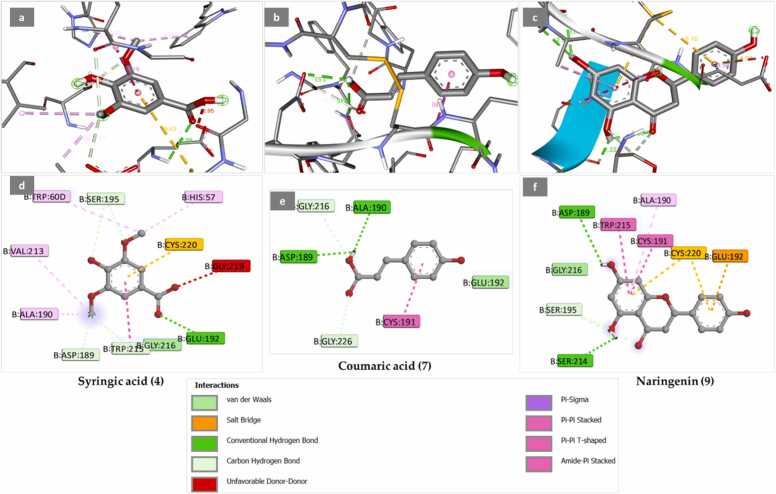
(a, b, c) the 3D binding mode of syringic acid (4), coumaric acid (7), and naringenin (9) within active pocket of the thrombin inhibitor complex (PDB: 1KTS); (d, e, f). the 2D binding mode of syringic acid (4), coumaric acid (7), and naringenin (9) within the active pocket of the thrombin inhibitor complex (PDB: 1KTS).

The docking result of the studied compounds revealed that syringic acid, coumaric acid, and naringenin showed good binding mode within the active pocket of the thrombin inhibitor complex (PDB: 1KTS) through the formation of multiple interactions. Table (4) shows the binding energy score of these four components towards the active pocket of the thrombin inhibitor complex along with the type of interactions. On the other hand, Fig. (3a-c) illustrates the 3D configurations of these components towards the active pocket of the thrombin inhibitor complex. Whereas, Fig. (3d-f) provides the 2D configurations of the four components towards the active pocket of the thrombin inhibitor complex.

**Table 4:** Binding energy (Kcal/mol) and interactions of the methanolic extract constituents inside the active pocket of the thrombin inhibitor complex (PDB: 1KTS).

Methanolic extract components	Binding energy Kcal/mol	Moieties from the compound	Amino acid residues	Type of interaction
Syringic acid	−5.9	CO	GLU192	Van der Waals
		O-CH_3_	ASP189, TRP215, SER195	Conventional H-bond
		Phenyl	CYS220	Pi-sulfur
		O-CH_3_	TRP60D, HIS57, VAL213, ALA190	Pi-Pi
		CO	GLY219	Unfavorable D-D
Coumaric acid	−5.7	O-CH_3_	ASP189, ALA190	Van der Waals
		O-CH_3_, CO	GLY216, GLY226	Conventional H-bond
		Phenyl	CYS191	Pi-Pi
Naringenin	−7.8	O-CH_3_	ASP189, SER214	Van der Waals
		O-CH_3_, CO	SER195	Conventional H-bond
		Phenyl	CYS220	Pi-sulfur
		Phenyl	TRP215, ALA190, CYS191	Pi-Pi
		Phenyl	GLU192	Salt-bridge

## 4 Discussion

The primary goal of this work was to assess the anticoagulant activity of several *C. speciosus* extracts, both *in vitro* and *in vivo*. The blood coagulation system is essential for controlling bleeding and maintaining homeostasis. Coagulation is an essential causal factor in disorders like ischemia, stroke, and COVID-19 [2], [26]. The link between hypercoagulation and cancer is well documented, and severe COVID-19 pneumonia causes many prothrombotic alterations [27]. Drugs that can treat cancer and have potential antithrombotic/anticoagulant properties would be desirable, especially if they are plant-derived. Plants are a safe medication source for preventing clotting. In this study, *C. speciosus* extracts were investigated for the first time to identify alternatives to dangerous anticoagulants, as the necessity for these anticoagulants increased as the number of lethal viral illnesses in the world increased. Our earlier investigation found that the extract of *C. speciosus* has substantial anticancer properties against the HePG2 cell line [16]. Blood coagulation is an essential component of hemostasis [28], and PT, aPTT, and TT are considered blood coagulation indicators [29]. PT is used to estimate coagulation factors V, VII, and X in the extrinsic pathway, whereas aPTT is used to evaluate coagulation factors VIII, IX, XI, XII, and prekallikrein in the intrinsic coagulation pathway of the coagulation cascade. The regular PT ranges from 12.5 to 13.7 s, while the aPTT ranges from 31 to 39 s. The PT is the standard test for monitoring coumarin therapy (vitamin K antagonists), whereas the aPTT is typically used to assess the efficacy of heparin therapies [30].

In the current work, the *in vitro* and *in vivo* results of aPTT, PT, and TT assays revealed that the SCME considerably delayed PT, TT, and aPTT clotting times in a dose-dependent manner. Meanwhile, the potential mechanism of SCME is probably through direct inhibition of the common pathway by reducing the generation of thrombin (factor IIa) through inhibiting factor Xa and its cofactor Va, or by blocking the thrombin interaction with substrate fibrinogen and thus preventing the formation of fibrin. These findings suggested the presence of a protease inhibitor in *C. speciosus*, blocking the conversion of zymogens into active factors Xa, Va, and thrombin. The active component(s) of *C. speciosus* most likely activate the natural anticoagulant pathway by binding to antithrombin III, causing a conformational change that can lead to the activation of protein C (PC) to activated protein C (APC), which conjugates its cofactor (protein S) and inactivates factors Va and VIIIa (cofactors for activation of factors Xa and IIa). This eventually blocks the activation of thrombin and factor Xa in the same route.

Heparin, warfarin, all-trans retinoic acid, and recently developed novel anticoagulants (NOACs) are common anticoagulant medications used to treat thrombotic disorders. Heparin is a short-acting (fast) reversible anticoagulant; warfarin is long-acting and sluggish, with reversible effects; all-trans-retinoic acid is slow-acting; and NOACs are fast and cannot be reversed [31]. Heparin exerts its anticoagulant action by attaching to a specific position of antithrombin and generating a conformational change that exposes the site that binds and inactivates thrombin (IIa) and factor Xa, hence enhancing antithrombin's anticoagulant activity by roughly 1000 times. Warfarin is a vitamin K antagonist that prevents the activation or synthesis of vitamin K-dependent proteins involved in the coagulation pathway (factors X, IX, VII, and prothrombin). Vitamin K acts as a cofactor for the carboxylase enzyme, which converts glutamic acid residues in vitamin K-dependent proteins to γ-carboxylates. Additionally, these proteins require γ-carboxylation of their glutamic acid residues for calcium binding and physiological activation [32]. Previous research has shown that all-trans retinoic acid has an anticoagulant effect by downregulating tissue factors and upregulating thrombomodulin expression, hence boosting the antithrombotic capability of microvascular endothelial cells. Although numerous treatments have been produced over time, the majority of them are associated with negative side effects [33]. The CSME displayed a restricted concentration range of activity peak, indicating a rapid and reversible action, comparable to heparin. This lends credence to the postulated mechanism of suppressing the co-clotting pathway, which is similar to heparin's. Several studies have demonstrated the anticoagulant effects of phenolic substances, including phenolic acids and flavonoids, which exhibit very modest thrombolytic action in a dose-dependent manner [34].

Previous research has confirmed the anticoagulant effect of several plants, such as *Melastoma malabathricum* Linn. (Aqueous leaf extract), which demonstrated a prolonged coagulation time [35]. Furthermore, it was proposed that the leaf extracts of *Sutherlandia frutescens*, *Gloriosa superba*, *Zantedeschia aethiopica*, and *Leonotis leonurus* had anticoagulant characteristics [36]. Coumarin produced from several plants, has been used as an anticoagulant because it blocks calcium action in the blood coagulation cascade. As a result, the antithrombotic effect of the examined plant extracts is most likely owing to their high phenolic and coumarin content, and the considerable rise in PT and aPPT is caused by phenolic compounds found in *C. speciosus* extracts interfering with calcium's procoagulant activity.

Furthermore, Adiba et al. [37] reported that flavonoids have thrombolytic action, and quercetin, which is primarily a strong free radical terminator, can reduce atherosclerosis, which leads to stroke and heart attack [38]. Thus, the anticoagulant properties of CSME may be attributed to the high content of these elements, which supports its traditional use in cardiac patients. The TT demonstrated thrombin inhibition-dependent clotting time, which was caused by fibrin polymerization. TT represents the blood coagulation status that converts fibrinogen to fibrin, which is directly induced by the addition of thrombin. In a recent study, we found that the methanolic extract of *C. speciosus* contained the most flavonoids and polyphenols, followed by the ethanolic and aqueous extracts [16]. The methanolic extract was high in quercetin, gallic acid, caffeine, cinnamic acid, gallic acid, and chlorogenic acid. These findings revealed that the anticoagulant action of these extracts was related to their high polyphenol and flavonoid content, as well as their ability to boost the efficiency of the CSME as compared to other extracts. Moreover, in another work, we identified six bioactive components using GC-MS [22] (Table 2S, Supplementary materials). These compounds demonstrated a variety of biological properties, including antioxidant, anti-parasitic, anti-acetylcholine, anti-tumor, anti-AD; and bone anabolic agent [39], [40], [41], and maybe contributed to the anticoagulant activity of the CSME.

Coagulant inhibition by the main bioactive components of the CSME may occur by preventing the interaction of thrombin with the substrate fibrinogen and thus preventing fibrin formation such as dabigatran (a potent, synthetic, reversible, non-peptide thrombin inhibitor). Inhibition of thrombin by dabigatran results in decreased fibrin formation and reduces thrombin-stimulated platelet aggregation and thus prevents the formation of blood clots [42], [43], [44]. Thus, in the present study, we investigated the interaction of the CSME constituents with the target thrombin inhibitor complex (PDB: 1KTS) using molecular docking analysis [45]. The original ligand dabigatran (C24) reproduced the interaction with the key amino acid ASP189 (the most important specific binding sites) of the active cave of the thrombin inhibitor complex (PDB: 1KTS) through a hydrogen bond as previously reported [46] confirming the validity of the docking process. Although, all the tested compounds were slid into the same position as the original ligand, three compounds out of thirteen, namely syringic acid, coumaric acid, and naringenin interacted with the key amino acid ASP189 (which is necessary for the inhibition of thrombin activity) of the active pocket of the thrombin inhibitor complex (PDB: 1KTS) in the same manner of the original ligand.

## 5 Conclusion

*C. speciosus* extracts have anticoagulant properties and prolong the activated partial thromboplastin time (aPTT), prothrombin time (TT), and thrombin time (PT) in a dose-dependent manner. However, the methanol extract was more potent, so we tested it *in vivo*. This extract demonstrated good results and may be used as a natural anticoagulant medication. The overall results of molecular docking simulation showed that the methanolic extract is a potential anticoagulant candidate capable of blocking the interaction of thrombin with its substrate fibrinogen and thus preventing fibrin formation. This may be due to the effect of its active compounds syringic acid, coumaric acid, and naringenin, and their effectiveness in binding to the main amino acid ASP189 of the thrombin inhibitor complex (PDB: 1KTS). To the best of our knowledge, this is the first study to investigate the anticoagulant activity of *C. speciosus* extracts *in vitro* and animal models.

## Ethics statement

This study received approval for the healthy volunteer donors from the ethics committee of Ferhat Abbes University, Steif 1, Algeria (ethical approval # D01N01UN190120120005), and for the animal study from the National Research Centre's Animal Care and Use Committee (ethical approval number 13050302/2022).

## Funding

This work was supported by Ferhat Abbes University, Setif 1, Algeria and the 10.13039/100007787National Research Centre, Dokki, Cairo, Egypt project # 13050302.

## CRediT authorship contribution statement

**Abdel-Wahhab Mosaad A.:** Writing – review & editing, Validation, Supervision, Funding acquisition. **El-Nekeety Aziza A.:** Methodology, Investigation, Data curation. **El-Sawy Eslam R.:** Visualization, Software, Methodology. **Noureddine Belattar:** Visualization, Software, Project administration. **Hassan Marwa E.:** Software, Methodology, Investigation, Data curation. **Gheraibia Sara:** Methodology, Investigation, Formal analysis.

## Declaration of Competing Interest

The authors declare that they have no known competing financial interests or personal relationships that could have appeared to influence the work reported in this paper.

## Data Availability

Data will be made available on request.
