# Supplementary material for: Molecular docking of polyphenol compounds and exploring the anticoagulant activity of Costus speciosus extracts in vitro and in vivo
Source: Toxicol Rep. 2025 Feb 19;14:101961. doi: 10.1016/j.toxrep.2025.101961 (PMC11908605; doi:10.1016/j.toxrep.2025.101961)
Supplement: Supplementary file 1 — Supplementary material [file mmc1.docx]

**Table 1S.** HPLC analysis of the total polyphenols of the three extracts of *C. speciosus^*^*

| polyphenol | Concentration (µg/g) | | |
| --- | --- | --- | --- |
|  | Aqueous | Ethanol (70%) | Methanol (70%) |
| Gallic acid | 2470.21 | 747.58 | 689.73 |
| chlorogenic acid | 590.55 | 1206.48 | 1175.07 |
| Caffeic acid | 154.29 | 79.25 | 92.86 |
| Syringic acid | 0.00 | 115.10 | 136.22 |
| pyro catechol | 306.19 | 300.34 | 371.77 |
| Ellagic acid | 139.69 | 193.28 | 265.48 |
| Coumaric acid | 0.00 | 57.58 | 125.92 |
| Vanillin | 0.00 | 0.00 | 40.29 |
| Naringenin | 873.80 | 1025.17 | 1069.65 |
| Propyl Gallate | 68.51 | 65.17 | 93.79 |
| 4`.7-DihydroxyisoFlavone | 55.33 | 46.77 | 100.54 |
| Quercetin | 172.35 | 0.00 | 127.65 |
| Cinnamic acid | 59.00 | 0.00 | 49.51 |

#### ^*^After Ghariba et al. (2020), <https://doi.org/10.1016/j.sajb.2020.02.019> (Open Access)

**Table 2S.** Chemical constitutes of *C. speciosus* extract identified by GC-MS^*^

| Peak | RT | Name | Formula | Area | Area Sum % |
| --- | --- | --- | --- | --- | --- |
| 1 | 22.71 | 2(3H)-Benzofuranone, 6-ethenylhexahydro-6-methyl-3-methylene- 7- (1-methylethenyl)-[ 3aS-3a.alphha., 7-a beta.)] | C_15_H_20_O_2_ | 2525779.85 | 12.64 |
| 2 | 23.208 | 2,5-Octadecadiynoic acid, methyl ester | C_19_H_30_O_2_ | 601648.85 | 3.01 |
| 3 | 23.791 | Dihydrodehydrocostus lactone | C_15_H_20_O_2_ | 2664171.64 | 13.33 |
| 4 | 24.283 | Azuleno[4,5-b] furan-2(3H)-one, decahydro-3,6,9-tris(methylene)-, [3aS-(3a. alpha.,6a. alpha.,9a. alpha.,9b. beta.)]- | C_15_H_18_O_2_ | 13207137.69 | 66.1 |
| 5 | 24.867 | 5,8,11,14,17-Eicosapentaenoic acid, methyl ester, (all-Z)- | C_21_H_32_O_2_ | 239544.12 | 1.2 |
| 6 | 25.073 | 5,8,11,14-Eicosatetraenoic acid, methyl ester, (all-Z)- | C_21_H_34_O_2_ | 743139.53 | 3.72 |

**After Ghariba et al. (2022),** [**https://doi.org/10.1016/j.toxicon.2022.05.002**](https://doi.org/10.1016/j.toxicon.2022.05.002)

**with permission from the publisher (permission number 5793051418702)**
